# Epigenetic tuning of tumour-associated macrophages (TAMs): a potential approach in hepatocellular carcinoma (HCC) immunotherapy

**DOI:** 10.1017/erm.2024.9

**Published:** 2024-09-25

**Authors:** Israa M. Helal, Monica A. Kamal, Mostafa K. Abd El-Aziz, Hend M. El Tayebi

**Affiliations:** Clinical Pharmacology and Pharmacogenomics Research Group, Department of Pharmacology and Toxicology, Faculty of Pharmacy and Biotechnology, German University in Cairo - GUC, Cairo, Egypt

**Keywords:** epigenetics, hepatocellular carcinoma (HCC), immunotherapy, macrophages, tumour-associated macrophages (TAMs)

## Abstract

Recent development in immunotherapy for cancer treatment has substantiated to be more effective than most of the other treatments. Immunity is the first line of defence of the body; nevertheless, cancerous cells can manipulate immunity compartments to play several roles in tumour progression. Tumour-associated macrophages (TAMs), one of the most dominant components in the tumour microenvironment, are recognized as anti-tumour suppressors. Unfortunately, the complete behaviour of TAMs is still unclear and understudied. TAM density is directly correlated with the progression and poor prognosis of hepatocellular carcinoma (HCC), therefore studying TAMs from different points of view passing by all the factors that may affect its existence, polarization, functions and repolarization are of great importance. Different epigenetic regulations were reported to have a direct relation with both HCC and TAMs. Here, this review discusses different epigenetic regulations that can affect TAMs in HCC whether positively or negatively.

## Introduction

Malignant tumour biology has been evolving for thousands of years, and cancer is considered the second leading cause of death worldwide. The global cancer burden is significant and increasing. According to the World Health Organization, by 2040 the expected number of cancer cases per year globally will be 29.4 million cases (Ref. 1). Cancer is considered as a group of diseases that can attack any type of tissue in the body without any alarm. The turning point for any cell that can lead to a malignant tumour is when a normal cell is genetically altered, for instance, mutations in oncogenes or tumour-suppressor genes. Besides, it can be epigenetically altered (change in DNA methylation and histone acetylation). These modifications cause cells to divide uncontrollably whether by increasing cell division rates and/or inhibiting apoptosis, leading to cell-cycle arrest. Cancer cells ensure their survival by diverse mechanisms (such as metastasis, angiogenesis, drug resistance, metabolic adaptation, reprogramming and cancer stem cells that can renew outbreaks of the disease). Over the past few years, myriad treatments were developed to cure cancer including surgeries, chemotherapy, radiotherapy, phototherapy and immune therapy. However, most of these treatments failed because of the complicated tumour progression and survival mechanisms which involve many variable factors (Ref. 2).

Liver cancer is one of the most aggressive types of cancers with high incidence rates that have tripled since 1980, while the death rates doubled since that time (Ref. 3). Liver cancer may be primary (develops in liver cells) or secondary (occurs as a result of metastasis of another type of cancer in the body) (Ref. 4). There are four subtypes of liver cancer: hepatocellular carcinoma (HCC), cholangiocarcinoma, liver angiosarcoma and hepatoblastoma (Ref. 5). HCC is the most common type of primary liver cancer. It accounts for approximately 90% of the cases (Ref. 6). HCC aetiology is mainly a result of liver diseases. The leading causes of liver disease are mainly hepatotropic viruses (hepatitis B (HBV) and/or hepatitis C (HCV)), metabolic disorders (morbid obesity, diabetes mellitus (Ref. 2), insulin resistance and hypertriglyceridaemia), genetic diseases (haemochromatosis, Von Gierke's disease, hepatic porphyrias and tyrosinaemia type I) and other factors (heavy alcohol and tobacco consumption). All of which eventually lead to cirrhosis which increases the risk of HCC by 30-fold (Ref. 7).

Multiple treatments have been developed, and currently being used for treating HCC include: surgical therapies (resection, cryoablation and liver transplantation) and non-surgical therapies, which may be liver-directed (i.e. percutaneous ethanol injection, radiofrequency/microwave ablation, trans-arterial embolization, external beam radiation therapy) or systemic (chemotherapy, molecularly targeted therapy) (Ref. 8). Yet survival rates are not that high and the long-term prognosis is quite poor (Ref. 2). Recently, targeting the host immunity has gained genuine attention to boost its role in attacking the tumour (immunotherapy). The cells of the immune system (white blood cells) are categorized into lymphocytes (T-cells, B-cells and natural killer (NK) cells), neutrophils and monocytes/macrophages (Ref. 9). The major proteins of the immune system are predominantly signalling proteins (often called cytokines), antibodies and complement protein (Ref. 10). White blood cells/leucocytes along with lymphocytes play a key role in the immune system's anti-tumour response (Ref. 9). However, in cancer, the cancerous cells switch some of the functions of the immune compartments by releasing signals and mediators in the tumour microenvironment (TME). Innate immune cells such as macrophages (Ref. 11), neutrophils (Ref. 12) and dendritic cells (DCs) (Ref. 12) can respond to these mediators and polarize to different forms (tumour-associated cells). These cells help in tumour progression and survival; as an example when a macrophage, a mononuclear cell, is polarized to tumour-associated macrophage (TAM), it shows broad effects on tumourigenesis (Refs 13, 14).

Lately, studies were concerned with studying TAMs, including their regulation with respect to epigenetic modulations. Epigenetic modulation reports did not only include DNA methylation and histone modification but also included how different long non-coding RNAs (LncRNAs) are regulated by cancerous cells and how these regulations affect TAMs (Ref. 15). In this review, we will discuss multi-faceted regulations controlling TAMs since TAMs represent a potential target for tumour diagnosis and treatment.

## Methods

To achieve the purpose of the review, a search was conducted at the States National Library of Medicine (PubMed) and Google Scholar. For the search in databases, the descriptors used were: ‘Hepatocellular carcinoma’/‘liver cancer’ and ‘Macrophages’, ‘TAMs’, ‘epigenetic regulations’, ‘long non-coding RNAs’, ‘miRNAs’, ‘histone modifications’ and ‘DNA methylation’. Research papers, books and published data were reviewed for their relevance to the aim of the review and summarized. Criteria for inclusion were complete, relevant publications, available online, in English, published between 2010 and 2021, with detailed information about participants, methods and analyses. Data collection was done during September/October 2020, and data abstracted was in the form of descriptive information, covering the type of samples used, techniques and findings or effects reported. Bias was limited through the evaluation of the studies through their internal validity rather than the conclusion.

## Results

### Immunotherapy and TME

It is widely believed that cancer cells do not function and progress alone. They co-operate with various types of immune cells (Ref. 16), mediators (Ref. 17), stromal cells (Ref. 18) and extracellular matrix (Ref. 19). Many immune cells were highlighted to assist in controlling immune responses that play a vital role in cancer progression, behaviour and survival, such as DCs (Ref. 16), cytotoxic T lymphocytes, NK cells (Ref. 20), cytokine-induced killer cells (Ref. 21) and macrophages (Ref. 11). TME, the area surrounding any solid tumour, is a complex cellular ecosystem that is regarded as an important part of tumour immunology because of its crucial role in tumour progression (Ref. 22). It is a colony of different cells including the surrounding blood vessels, fibroblasts, cytokines, growth factors and a variety of immune cells (such as T cells, B cells, macrophages, DCs and mast cells) (Ref. 23). These cells are steadily modified during the development of cancer, which in turn provide strong support to the tumour cells in cancer formation, progression and surviving through different ways including proliferation, invasion, angiogenesis, metastasis (Ref. 24), immune tolerance (Ref. 25) and drug resistance of tumour cells (Ref. 26). In addition, TME is characterized by lactate accumulation (because of high-glucose consumption) that causes low pH ranging from 6 to 6.5 (that is favourable for the metastasis, angiogenesis, and, more vitally immunosuppression) (Ref. 27). Unfortunately, the tumour cells started to develop different pathways and techniques that allow them to recruit immune cells and immune checkpoints for their advantages (Ref. 22). Accordingly, the immunotherapy approach, which is considered as the fourth pillar for cancer treatment and had shown unprecedented results with patients with different cancer types, is based on two main aspects: the first is controlling the highlighted immune cells involved in TME to yield the expected anti-tumour effect, and the second aspect is studying the different TME pathways controlling these cells (Ref. 28). One of the vital immune cells that are recruited to TME is macrophages. Macrophages are highly infiltrative in TME; they are recruited first to TME, then they are polarized to TAMs and here macrophages start their roles as anti-tumour suppressors (Ref. 29).

### Macrophages

Macrophages, one of the most plastic cells of the haematopoietic system, are originated from mononuclear cells derived from bone marrow, embryonic sac and post-natal myeloid or foetal liver and they are found in all tissues and have exceptional functional diversity (Ref. 30).

Normally, macrophages are recruited to infected sites or injury sites in order to start their role in invading pathogens through several functions (Ref. 31). In recruiting sites, macrophages do not only recruit other immune cells by secreting several mediators, including cytokines and chemokines but also work by other mechanisms (Ref. 32). They promote tissue repair by producing factors (angiogenesis factors, growth factors and proteases) (Ref. 31), then they use nitrogen free radicals and reactive oxygen species to kill the pathogen (Ref. 31). In the end, they may present extrinsic antigens to cytotoxic T-cells (Ref. 33).

**Figure 1.**
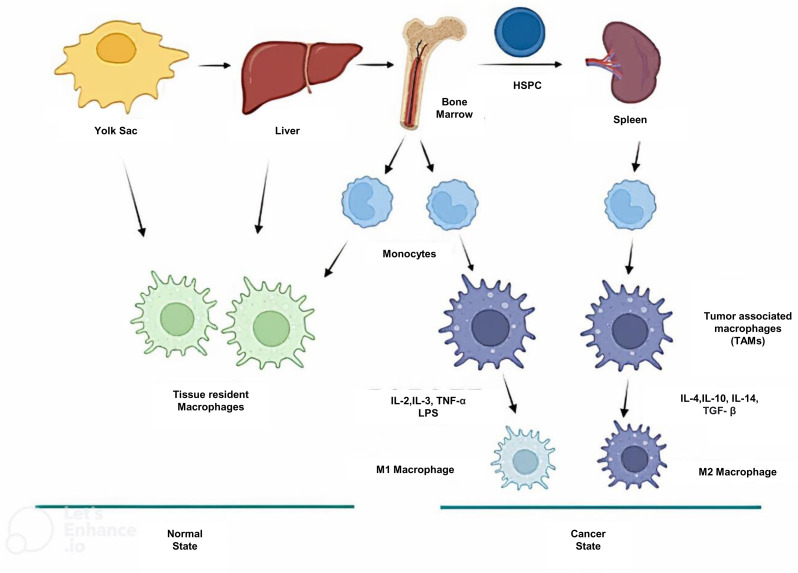
Origins of tissue-resident and TAMs. Tissue-resident macrophages are mainly derived from yolk sac during development. TAMs are derived from tissue-resident macrophages, or by differentiation of monocytes from the bone marrow. TAMs are polarized into M1-like or M2-like phenotypes based on signals received from the TME. HSPCs, haematopoietic stem cells and progenitor cells.

Macrophages can transduce signals received from various sources in the tissue environment to promote homoeostasis on behalf of its ability to reach any cell in the body through their release to the circulating bloodstream (for hours or days). In cancer, macrophages act as a double-edged sword. As macrophages can be activated into two distinct subsets based on the M1/M2 model, classically activated or M1 macrophages and alternatively activated or M2 macrophages (Ref. 34) (Fig. 1).

### Tumour-associated macrophages

#### TAM polarization and recruitment

Here, the role of TME in the conversion of macrophages to TAMs will be discussed in terms of recruitment and polarization. Various recruiting factors are responsible for monocytes' efflux and macrophages' recruitment, counting the CC-chemokine ligands (CCL2 (Ref. 35), CCL5 (Ref. 36) and CCL18 (Ref. 37), chemokine receptor (CXC) chemokine family (IL-4, IL-10), CXC-chemokine ligands (CXCL1, CXCL6 and CXCL8) (Ref. 38), vascular endothelial growth factor (VEGF), platelet-derived growth factor, hypoxia-inducible factor (HIF) (Ref. 39) and transforming growth factor-*β* (TGF-*β*) (Ref. 40), cerebrospinal fluid (CSF)-1 (Ref. 41) and nicotinamide-adenine dinucleotide phosphate (NADPH) oxidase 4 (NOX4) (Ref. 42). It is conveyed that the serum level of CCL2 is higher in some cancers, suggesting that CCL2 also has a role in macrophages; recruitment, and infiltration in TME, as CCL2 binds specifically to CCR2 that is highly expressed in macrophages (Ref. 35).

After the recruitment, macrophages face the phase of polarization. They can be polarized whether to classically activated macrophage (M1) or alternatively activated macrophage (M2) phenotype depending on the influencing factors they receive (Ref. 43). M1 is polarized when exposed to microbial products (e.g. lipopolysaccharides) or pro-inflammatory cytokines (e.g. interferon -*γ*, IL-2, IL-3, IL-12, tumour necrosis factor (TNF)-*α* or Toll-like receptor (TLR) ligands). M1-like polarized macrophages work with anti-tumour immunity and inflammatory responses since they are responsible for the production of pro-inflammatory factors such as IL-1 beta, IL-6, IL-12, IL-23, inducible nitric oxide synthase (iNOS) and TNF-*α*, chemokine ligands 9 (CXCL-9), CXCL-10 and express the major histocompatibility complex class I and class II (MHC II) molecules (Ref. 43). M1 has specific surface markers (CD80, CD86, TLR-2, TLR-4) (Ref. 44).

**Figure 2.**
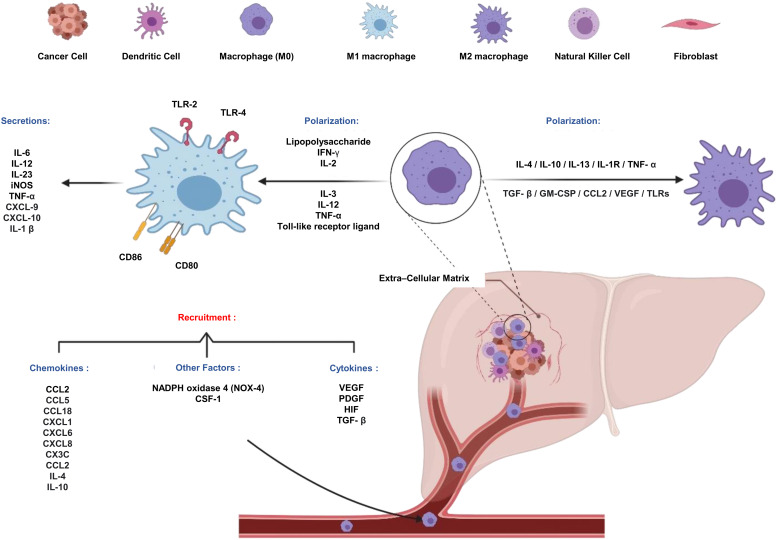
Recruitment and polarization of macrophages. (1) TME different types of cells (DCs, macrophages, NK cells, fibroblasts). (2) Recruiting factors of macrophages towards TME, (3) Diverse polarization factors that affect the polarization of macrophages whether to M1 or alternatively M2, (4) Markers of M1, (5) Secretions of M1.

Different factors affect TAM polarization. M2 are polarized through the exposure of macrophages to Th2 cytokines such as IL-4, IL-10, IL-13, interleukin-1 receptor (IL-1R), TNF-*α*, TGF-*β*, granulocyte macrophage colony-stimulating factor (GM-CSF), CCL2, VEGF, immune complexes (ICs) and TLRs (Ref. 43) (Fig. 2). Another factor is hypoxia which is one of the TME characteristics. Hypoxia enhances the attraction of macrophages towards the tumour and decreases M1 polarization (Ref. 45). Several studies suggested that chronic alcohol consumption associated with HCC can cause an increase in TAM density since diethyl-nitrosamine is promoted which was confirmed to induce immune disturbance (Ref. 46). Conversely, M2-like macrophages produce various anti-inflammatory molecules such as IL-10, TGF-*β*, glucocorticoid hormones and arginase-1 and exert pro-tumourigenic activities (Refs 47, 48). M2 macrophages highly produce CCR2, CXCR1 and CXCR2 (Refs 48, 49), that play crucial roles in the growth, angiogenesis (Ref. 50), metastasis (Ref. 51), invasion (Ref. 52) and drug resistance (Ref. 53) of malignant melanomas. In addition, they produce dendritic cell-specific intercellular adhesion molecule-3-grabbing non-integrin (DC-SIGN) (cluster of differentiation 209, responsible for DC recruitment) (Ref. 54), CD163, CD209 and CD206 which are the most common surface markers used to identify M2 phenotype (Refs 55, 56). Recent studies showed that M2-like phenotypes are mostly considered TAMs (Refs 57, 58, 59).

Recent studies have reported that mitochondrial DNA (mtDNA) stress can regulate innate immunity (Ref. 60). mtDNA stress is defined as broad modulations in nuclear gene expression because of different metabolic stimuli and other changes within mitochondria. These modulations are generally stated as mitochondrial stress responses (Ref. 61). mtDNA stress can be achieved by different pathways including alterations in mitochondrial nucleotide structure leading to the release of mtDNA in the cytosol (Ref. 60). Drp-1 is one of the key proteins for mitochondrial fission, which plays an important role in HCC progression and survival. A study published in 2019 investigated the relation between Drp-1 protein and CD163 (one of the most common markers for both M2 and TAMs). The findings showed that (1) initially it was shown that Drp-1 expression was positively correlated with the existence of CD163 positive cells in HCC. Moreover, HCC patients with high Drp-1 expression had a significantly poor overall survival. (2) Drp-1 overexpression considerably increased mRNA expression and protein secretion of CCL2. This increase was done through the TLR9-mediated nuclear factor kappa B (NF-*κ*B) signalling pathway that in return increased TAM recruitment and polarization. To sum up, Drp-1 causes cytosolic mtDNA stress leading to an increase in the expression of CCL2 that enhances TAM polarization and recruitment (Ref. 62) (Fig. 2).

#### TAM subtypes, roles and cytokines in HCC TME

One of the oncogenic techniques that cancer cells follow is secreting the factors that promote M2 polarization, and it is reported that they shift M1 phenotype to M2 phenotype as well (Ref. 63). M2 macrophages can be further classified into four subtypes each with its unique functions, and mediators. They are classified as follows: M2a type, induced by IL-4 or IL-13 (promotes tissue repair through the secretion of extracellular matrix (ECM)); M2b type, induced by exposure to ICs and agonists of TLRs or IL-1R (participates in anti-inflammatory responses and functions in immune regulation); M2c type, induced by glucocorticoid hormones and IL-10 (suppresses immune responses and tissue remodelling). TAM polarization and densities were associated with tumour progression and poor prognosis in HCC patients. Lastly, M2d, induced by the TLR antagonists (release of IL-10 and VEGFs and promotion of angiogenesis and tumour progression) (Ref. 44).

TAMs secrete a series of cytokines and inflammatory factors such as TGF-*β*), IL-6, IL-10, IL-13, IL-4 and TNF-*α* that promote tumour initiation, growth, metastasis, angiogenesis, proliferation and chemoresistance in HCC (Ref. 64) (Fig. 2). During metastasis, TAMs also stimulate HCC tumour cell extravasation, survival and insistent growth. They have critical roles in tumour development, such as (1) promotion of angiogenesis by releasing VEGF, which promotes blood vessel anastomosis (Ref. 65), regulation of the expression of CXCR4 via the extracellular signal-regulated kinase (ERK) pathway (a novel vascular marker for angiogenesis in HCC tissues) (Ref. 66). (2) Promotion of HCC cells' proliferation, invasion and metastasis by releasing potent IL-1*β* in the TME leading to upregulation of HIF-1*α* synthesis which in return enhances epithelial–mesenchymal transition (EMT) of hepatoma cells (Ref. 67). In addition, TAMs can enable the migration and EMT of HCC cells through the TLR4/signal transducer and activator of transcription 3 (STAT3) signalling pathway (Ref. 68). (3) Indirect promotion of a pro-oncogenic inflammatory microenvironment and tumour invasion of HCC cells and EMT after being induced by abnormal activation of nucleus tractus solitarius (NTS)/IL-8 pathway (Ref. 69). (4) Promotion of EMT and cancer stemness of liver cancer stem cells through the Wnt/*β*-catenin pathway (Ref. 70). (5) Controlling the observed therapeutic resistance to trans-arterial chemoembolization (TACE) where evidence-based studies showed that the density of TAMs in HCC was related to the efficacy of TACE (Ref. 14). Another study performed on the effect of TAMs on oxaliplatin resistance showed that TAMs affect the resistance to the drug by inhibiting apoptosis in HCC through induction of autophagy (Ref. 13). (6) Secretion of several cytokines and chemokine recruiting regulatory T cells into the TME, inhibiting the function of CD4+ and CD8+ effector T cells leading to immunosuppression (Ref. 71) (Fig. 2).

#### Strategies aimed at targeting and regulating TAMs and TANs

TAMs have a pivotal role in the development, prognosis and treatment of HCC (Refs 1, 2). TAMs can manifest either anti-tumour (M1) or pro-tumour (M2) functions, and their presence in HCC is linked to heightened tumour growth and spread. Additionally, TAMs contribute to chronic inflammation, which fosters the progression of HCC. The neutrophil-to-lymphocyte ratio (NLR), indicative of tumour-associated neutrophils (TANs), has shown associations with unfavourable outcomes in HCC patients (Ref. 2). Strategies aimed at targeting and regulating TAMs and TANs have been explored as potential therapeutic approaches for HCC. A range of treatment methods, such as immunotherapy, small-molecule inhibitors, immune checkpoint inhibitors, antibodies, tumour vaccines, adoptive cellular immunotherapy and nanocarriers for drug delivery, have been studied to target TAMs and enhance the prognosis and overall quality of life for individuals with HCC (Refs 1, 3).

TAMs play a pivotal role in HCC and other solid tumours, promoting tumour growth and metastasis. TAMs can be either anti-tumour (M1) or pro-tumour (M2), and their polarization affects the TME and response to immunotherapies. Understanding TAM immunobiology is crucial for advancing HCC treatments. Researchers are investigating monocyte-targeted approaches to manipulate processes such as recruitment, activation and migration for potential HCC therapy innovations (Ref. 1).

TANs play a substantial role in the pathogenesis and progression of HCC. There are two distinct subsets: N1 TANs, which exhibit anti-tumourigenic properties, and N2 TANs, which are linked to heightened HCC growth, invasiveness and metastasis. The NLR is a dependable biomarker in HCC, with an elevated NLR associated with poorer patient prognosis. Clinical research is actively exploring various approaches centred on neutrophils to target and modulate processes such as recruitment, activation and migration, offering promising avenues for HCC therapy (Ref. 2).

The role of the tumour immune microenvironment in liver cancer, especially in nonalcoholic steatohepatitis-related HCC, remains poorly understood. Neutrophils, specifically known as TANs, are associated with unfavourable outcomes in HCC patients. Neutrophils display diverse characteristics and can impact the effectiveness of immune checkpoint inhibitor therapy. The combination of CXCR2 inhibitors with anti-PD1 (programmed cell death 1) treatments shows potential in transforming pro-tumour TANs into anti-tumour counterparts. Furthermore, the use of atezolizumab and bevacizumab together in advanced HCC holds promise, emphasizing the importance of targeting angiogenesis and regulating immune responses for enhanced liver cancer therapy (Ref. 3).

#### Epigenetic alterations affecting TAMs

Epigenetics refers to changes in gene expression levels based on non-gene sequence changes such as DNA methylation, histone modifications, chromosomal remodelling and non-coding RNA (ncRNA) regulation (Refs 72, 73). They mainly control the function and characteristics of genes by regulating the transcription or translation processes (Ref. 74). In addition, they also control and maintain various basic cellular processes (such as cell divisions, DNA repair, differentiation, apoptosis, angiogenesis, metastasis, growth factor response, detoxification and drug resistance) (Ref. 75). Although multiple studies have reported some of the epigenetic alterations affecting TAMs, unfortunately, the role of these alterations is not yet fully understood. Accordingly, TAM epigenetic tuning is a hallmark topic that attracts many researchers. The research conducted on TAMs focused on determining whether it is more effective to reduce the polarization of TAMs or to induce repolarization in order to mitigate the undesirable effects associated with TAMs. This investigation was initiated by studying the potential factors that influence the polarization of TAMs, with the aim of identifying strategies to decrease or eliminate these factors and subsequently reduce TAM polarization. This section discusses various epigenetic alterations that affect TAMs whether directly or indirectly.

### Long non-coding RNAs

**Figure 3.**
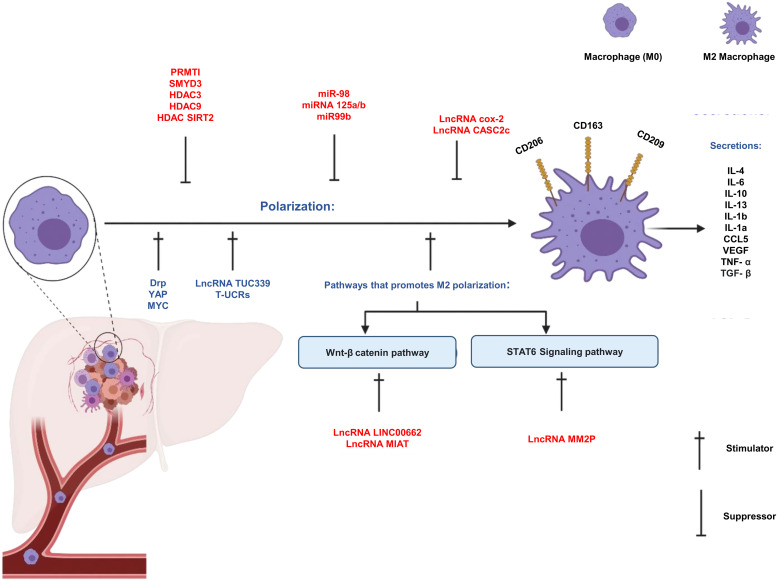
Regulation of TAM polarization. (1) Different types of stimulators and suppressors of M2/TAM polarization (including LncRNAs, miRNAs, proteins, pathways, etc.). (2) Cellular markers. (3) Some factors that are secreted from M2/TAMs.

The whole genome is divided into coding regions (transcribed into protein-coding RNA) and non-coding regions (transcribed into ncRNA). ncRNA is subtyped into linear RNA and circular RNA (Ref. 76). LncRNA is a type of linear non-coding RNA (more than 200 nucleotides). Several lines of evidence suggested that LncRNAs play an important role in tumour biology in terms of uncontrolled cell cycle, cell differentiation and have suggested to have a role in epigenetic alteration, mRNA stability and protein regulation. Therefore, studying LncRNAs affecting TAMs is crucial to understand TAMs' behaviour in TME. Many LncRNAs were reported to affect TAMs whether by enhancing or inhibiting the expression and functions or inhibiting of TAMs (Fig. 3). In the next section, the effect of multiple LnRNAs on polarization, epigenetic modulation and classic signalling pathways of TAMs will be discussed (Ref. 77).

#### Cyclo-oxygenase (cox)-2

LncRNA cox-2 played a major role in the regulation of inflammation responses (Ref. 78) in the various types of cancers (Refs 79, 80). The relations between LncRNA cox-2 and macrophage polarization reflecting the functions in HCC were investigated. This was performed by using lipopolysaccharides that induced polarization of macrophages to M1 phenotype and IL-4 that induced polarization of macrophages to M2/TAMs. Two mouse hepatic cell lines (Hepal-6 and HepG2) were selected and analysed using different techniques (quantitative real-time polymerase chain reaction (qRT-PCR), enzyme-linked immunosorbent assay, western blotting, 3-[4,5-dimethylthiazol-2-yl]-2,5 diphenyl tetrazolium bromide (MTT), colony formation assay, flow cytometry, transwell assay and stretch test). The results confirmed an inverse relation between LncRNA cox-2 expression and TAMs' polarization, as the study showed that M1 macrophages have a higher expression for LncRNA cox-2 than M2 macrophages and non-polarized macrophages. Silencing of LncRNA cox-2 caused an increased expression of M2 markers (Arg-1, IL-10 and Fizz-1) and a decreased expression of M1 markers including IL-12, iNOS and TNF-*α*. This demonstrates that suppression of LncRNA cox-2 decreases the polarization of macrophages to M1 and shifts the polarization to M2.

Regarding HCC, the study showed that the suppression of LncRNA cox-2 negatively affected the ability of M1 to inhibit proliferation, metastasis, angiogenesis, tumour formation and EMT. Moreover, it decreased the ability of M1 to promote apoptosis of HCC cells. On the contrary, suppression of LncRNA cox-2 positively strengthens the ability of M2 to promote proliferation, metastasis, EMT, angiogenesis, tumour formation and inhibition of apoptosis. These data verified that LncRNA cox-2 helps in the suppression of HCC immune evasion and progression by inhibiting the polarization of macrophages to TAMs besides enhancing the polarization of macrophages to M1 (Ref. 79) (Table 1).

**Table 1.** Summary of some epigenetical factors that affects TAM regulation

TAM regulator	Members	Model	Main findings	Effect	Ref.
Lnc-RNA	Cox-2	Hepal-6 and hepatoma cell line HepG2	LncRNA cox-2 siRNA decreased the expression of IL-12, iNOS and TNF-*α* in M1 macrophages, increased the levels of IL-10, Arg-1 and Fizz-1 in M2 macrophages. LncRNA cox-2 siRNA reduces the ability of M1 macrophages to inhibit HCC cell proliferation, invasion migration, EMT, angiogenesis and facilitate apoptosis. It also strengthens the ability of M2 macrophages to promote proliferation HCC cell growth and inhibit apoptosis.	Negative	79
	LINC00662	In vivo	LINC00662 upregulated WNT3A expression and secretion *via* binding miR-15a, miR-16 and miR-107. LINC00662 activated Wnt/*β*-catenin signalling in HCC cells in an autocrine manner and promoted HCC cell proliferation, cell cycle and tumour cell invasion, while repressing HCC cell apoptosis. It also promoted M2 polarization, HCC tumour growth and metastasis in vivo	Positive	81
	TUC339	In vitro THP-1 cells and HCC cells	LncRNA-TUC339 promotes the polarization of macrophages to M2/TAMs. It monitored pro-inflammatory cytokine production, co-stimulatory molecule expression and phagocytosis. It also suppressed cell motility and cell migration of THP-1 cells.	Positive	82
	CASC2c	In vitro HCC, GC and CRC cells	CASC2c overexpression inhibited the growth, migration and invasion of HCC cells in vitro and promoted their apoptosis. It decreased p-ERK1/2 levels in HCC, GC and CRC cells. Overexpression of CASC2c decreased *β*-catenin expression in HCC and GC cells; it increased that in CRC cells.	Negative	83
	CASC2c	GBM cell lines	LncCASC2c act as an inhibitor for coagulation factor X. LncCASC2c is considered as a tumour-suppressor agent	Negative	84
	MM2P	Cell-based M2 macrophage polarization models	LncRNA-MM2P was the only LncRNA upregulated during M2 polarization but downregulated in M1 macrophages. Knockdown of LncRNA-MM2P blocked cytokine-driven M2 polarization of and weakened the angiogenesis-promoting feature of M2 macrophages by reducing phosphorylation on STAT-6. Manipulating LncRNA-MM2P in macrophages impaired tumourigenesis, tumour growth in vivo and tumour angiogenesis.	Positive	85
	MIAT	BALB/c nude mice was used to establish HCC models	Upregulation of MIAT was directly associated with high level of histone H3 and H4 acetylation in HCC tissues compared with normal tissues. When MIAT was upregulated, HCC cell viability, proliferation and invasion were all enhanced. All these effects occurred because of MIATs' negative regulation on miR-214.	Positive	86
T-UCRs	uc.306	In vitro HCC tissues	uc.306 is involved in different pathways including Wnt pathway, Hippo signalling pathway and Hedgehog signalling pathway, which are known to affect M2/TAM polarization. uc.306 plays a role in HCC development through affecting HCC formation, growth, migration, metastasis and EMT.	Negative (it is downregulated in HCC cells)	87
miRNAs	miR-98	HCC cells HepG2 and SMMC7721	miR-98 mimic significantly suppressed the HCC-conditioned TAM-mediated promotion of cell migration, invasion and EMT in HepG2 and SMMC7721 cells. miR-98 inhibitor exerted reversed effects.	Negative	88
	miRNA 125a/b	In vitro	HCC cells were treated with TAM exosomes or transfected with miR-125a/b suppressed cell proliferation and stem cell properties by targeting CD90. The study indicated that miR-125a/b targeting CD90 played important roles in cancer stem cells of HCC.	Negative	89
	miR-99b	HCC or subcutaneous Lewis lung cancer (LLC) mice	TAMs significantly impeded the growth of HCC and LLC, especially after miR-99b delivery. miR-99b re-educated TAM towards anti-tumour phenotype with enhanced immune surveillance. miR-99b promoted M1 while suppressing M2 macrophage polarization by targeting *κ*B-Ras2 and/or mTOR, respectively.	Negative	90
Histone modulations	PRMT1	In vivo and in vitro	It exerts these effects through regulation of PPAR*γ* and methylation CIITA.	Positive regulator for M2. Decreases M1 polarization	91
	SMYD3	In vitro	It exerts this effect through H3K4 methyl transferase.	Positive regulator for M2 polarization	92
	HDAC SIRT2	In vitro	It promotes its effect on M2 through promotion of GATA3, Arg-1 and CD11C expression. It promotes its effect on M1 by downregulating of NF-κB signalling, IL-1B and TNF secretion.	Positive regulator for M2. Inhibits M1 polarization	93
	SIRT4	Homograft mouse model	It downregulated TAM FAO–PPAR*δ*–STAT3 axis	Negative regulator for M2 polarization	94
	HDAC3	In vivo and in vitro	It exerts these effects through inhibiting IL-4 signalling and TGF-*β* production. Opposing function by increasing the polarization of M1.	Negative regulator for M2 polarization Positive regulator for polarization of M1	95
	HDAC6	In vivo	Through regulating PD-L1 and other immunosuppressive pathways that are prompted by anti-PD-1 blockage treatments.	Negative regulator for M2 polarization Reprogram TAMs to an M1 phenotype	96
DNA methylation	DNMT3B	Macrophage–adipocyte coculture system	When DNMT3B is methylated, this negatively affects M2/TAM polarization. By modifying DNMT3B, M2 polarization was promoted unlike M1 polarization that was inhibited. Overexpression of DNMT3B downregulate IL-4-induced expression of Arg-1 contributing to downregulation of macrophage polarization.	Downregulation of macrophage polarization	97
	NOR1	HCC cell line HepG2 cells	NOR1 promotes M2 polarization. NOR1 was found to be silenced by hyper-methylation that can affect M2 polarization and associated with poor prognosis of the disease because of increased expression of iNOS.	Hyper-methylation decreased M2 polarization	98
Proteomics	YAP	In vitro co-culture systems	YAP activation is important in TAM recruitment towards TME in HCC via modulating the levels of IL-6, CSF-1 and CCL-2 secreted by the tumour cells. Induced the formation of tumour initiation cells.	Silencing YAP caused decrease in M2 levels	99
	MYC	In vivo	Promotes cell growth and differentiation. MYC likewise can control the expression of the tumour-promoting factors MMP9, VEGF, TGF-*β* and HIF-1.	Positive effect on tumour growth	100
Signalling pathways	Wnt/*β*-catenin	In vivo mouse models	Wnt/*β*-catenin signalling was activated during macrophage differentiation. Greatly expressed in M2-polarized macrophages.	Enhanced M2 macrophage polarization	101
	STAT-6	In vitro	Signalling pathway for both cytokines IL-4 and IL-13, which are related to TAMs’ polarization.	Negative Decreases tumour growth	102
	PI3K/Akt	In vitro	TGF-*β*, IL-10 and BMP-7 are regulated through PI3K/Akt. These signals are mainly responsible for enhancing M2 polarization. Akt activation is crucial in inducing IL-10 in macrophages through pro-inflammatory signals.	Enhances M2 polarization	101

#### LncRNA LINC00662

Long intergenic non-protein coding RNA 662 (LINC00662) is located in chromosome 19q11 (Ref. 79). LncRNA LINC00662 is diligently correlated with the existence and progression of different types of cancers such as osteosarcoma (Ref. 103), lung squamous carcinoma (Ref. 104), prostate cancer (Ref. 105), colon cancer (Ref. 106), oral squamous cell carcinoma (Ref. 107) and lately with HCC (Ref. 81). A study performed in 2019 concerned with the relation between LncRNA LINC00662 and HCC focusing on its effect on macrophages. It showed that LINC00662 binds physically to miR-15a, miR-16 and miR-107, which positively affect the activation of the Wnt/*β*-catenin signalling pathway and upregulation of WNT3A expression. Since Wnt/*β*-catenin signalling has an important role in HCC, thus studying the effect of LncRNA LINC00662 on HCC was crucial. Results confirmed that LncRNA LINC00662 promotes HCC cell proliferation, cell cycle, invasion and tumour growth.

Then, the effect of LncRNA LINC00662 on macrophages was highlighted, using three models of tris(hydroxypropyl)phosphine (THP)-1 (a human monocytic cell line) differentiated macrophages. The first model was treated with conditional medium from wild type, the second was treated with miR-15a/16/107 binding sites mutated LINC00662 stably overexpressed HCCLM3 cells and the last one was treated with conditional medium from LINC00662 stably silenced SK-HEP-1 cells. The results revealed that the cells treated with conditional medium from wild type showed an increase in Wnt/*β*-catenin signalling targeting genes (such as cyclin D and c-MYC). In addition, it showed an increase in M1 surface markers (such as IL-12, iNOS and TNF-*α*) and a decrease in M2 surface markers (such as CD163, IL-10, ARG1 and mannose receptor C-type 1 (MRC1)). However, the cells treated with conditional medium from LINC00662 stably silenced SK-HEP-1 cells revealed that cyclin D1 and c-Myc were downregulated. Moreover, M2 macrophage surface markers (such as CD163, IL-10, ARG1 and MRC1) were significantly increased.

Finally, these results of the last model strengthened the hypothesis that LINC00662 overexpression in HCC cells activates Wnt/*β*-catenin signalling and induces M2 macrophage polarization. To support this point, more THP-1 differentiated macrophages were treated with a Wnt signalling inhibitor (ICG-001) that also reversed the reduction of M1 markers and the increase of M2 surface markers. Collectively, it is suggested that expression of LINC00662 is increased in HCC tissues and associated with WNT3A expression, M2 macrophage polarization and poor outcome in HCC patients (Ref. 81) (Table 1).

#### LncRNA-TUC339

TUC339 is found with high density in HCC cells and is functionally involved in regulating tumour cell development and adhesion. When TUC339 was suppressed using siRNA, reduction in HCC cell proliferation, clonogenic growth and growth in soft agar were observed (Ref. 108). Later, a research group from Sichuan University focused on LncRNA-TUC339 and its effect on macrophages' functions and polarization. The study was initiated by testing HCC-derived exosomes (as several LncRNAs are higher in exosomes isolated from HCC cell line compared with that isolated from normal liver cell line) to figure out all its characteristics and encounter whether HCC-derived exosomes could possibly target macrophages (by incubating the exosomes isolated with THP-1 cells). The results showed that exosomes can be internalized by THP-1 cells (representing macrophages). After showing that LncRNAs can be internalized in macrophages, the study was concerned with the relation between LncRNA-TUC339, HCC and macrophages. The expression of LncRNA-TUC339 was first tested using two different cell lines: HCC cell line ‘PLC/PRF/5’ and normal liver cell line ‘HL-7702’. It was observed that LncRNA-TUC339 was highly expressed in both PLC/PRF/5 exosomes and THP-1 in PLC/PRF/5 cell line.

Then, the effect of TUC-339 on macrophage polarization was tested on two different macrophage cell lines transfected with TUC339 expression vector or empty control vector and treated with IFN-*γ* and lipopolysaccharide (LPS) or IL-4. Both experiments yield the same conclusion that LncRNA-TUC339 promotes the polarization of macrophages to M2/TAMs. In addition, the study showed how LncRNA-TUC339 affects the functions of macrophages by monitoring pro-inflammatory cytokine production, co-stimulatory molecule expression and phagocytosis. Also, by observing the pathways that are affected by knocking down LncRNA-TUC339, and how these pathways have a role in macrophages' polarization and functions. Knocking down of TUC339 showed an increase in both pro-inflammatory cytokine production and co-stimulatory molecule expression, enhanced phagocytosis and reduced cell viability. Phagocytosis, which is a principal feature of macrophages, can be efficiently initiated by Fc*γ* receptor (Fc*γ*R); TLRs and complement receptor-mediated ligand binding. Two obvious explanations for the enhanced phagocytosis monitored against TUC 339 were reported. The first was the Fc*γ*R-mediated phagocytosis pathway that was upregulated when TUC-339 was downregulated. The second was downregulation of the actin cytoskeleton pathway upon over-expression of TUC339 (Ref. 82). The actin cytoskeleton is responsible for the mechanical framework in order to achieve shape changes. In addition, it regulates signal transduction events (Ref. 109). Additionally, gene ontology (GO) analysis of the microarray data pointed out the suppression of cell motility and cell migration of THP-1 cells when TUC339 was overexpressed (Ref. 82) (Table 1).

#### LncRNA-CASC2c

LncRNA-CASC2c is located on chromosome 10q26. It is divided into three transcript subcategories CASC2a, CASC2b and CASC2c. CASC2c was first monitored in endometrial cancer, and then it was found to be downregulated in several types of cancers including HCC as it plays a role in tumour suppression (Ref. 110). A study investigated the expression of CASC2c on HCC cell lines and reported that it was downregulated. Then, the effect of CASC2c on cell proliferation, migration and invasion in HCC, gastric cancer and colorectal cancer was studied on three cell lines. Results have shown that CASC2c not only inhibits cell proliferation, migration and invasion, but also stimulates apoptosis. PcDNA3.1–CASC2c and empty pcDNA3.1 were transfected into the cell lines to achieve CASC2c overexpression. Results have shown that CASC2c overexpression meaningfully; inhibited cell proliferation, suppressed the migration and invasion and restricted growth (Ref. 83).

In addition, the effect of CASC2c on HCC cells was investigated. It initiated with monitoring the occurrence of CASC2c in HCC using various HCC cell lines and qRT-PCR; the results corroborated that LncRNA CASC2c was downregulated in HCC. Then the study focused on the effect of CASC2c on cell proliferation, migration and invasion in HCC, guanine-cytosine content (GC) and colorectal cancer (CRC). The findings suggested that CASC2c not only inhibits cell proliferation, migration and invasion, but also stimulates apoptosis. Three cell lines (but QGY-7703 was specific for HCC), were used in this study, pcDNA3.1–CASC2c and empty pcDNA3.1 was transfected into the cell lines, and then qRT-PCR was performed to confirm the overexpression of CASC2c. The results strongly suggest that LncRNA CASCc is downregulated in HCC (Ref. 83).

One more study was conducted on CASC2c with respect to TAMs, but this time was performed on glioblastoma multi-form (GBM) cell line. The outcomes revealed that coagulation factor X is highly expressed in TME. This factor is known to enhance both recruitment of macrophages and polarization of TAMs/M2. Coagulation factor X performs its' function through increasing the phosphorylation and activation of ERK1/2 (ERK can also enhance the angiogenesis of the tumour) and AKT in macrophages. After that, the study verified that LncCASC2c acts as an inhibitor for coagulation factor X, therefore LncCASC2c is considered as a tumour-suppressor agent (Ref. 84).

By summing up all the previous results, LncRNA CASC2c is downregulated in HCC and it is quite clear that LncCASC2c indirectly influences TAMs regarding their recruitment and polarization (Table 1).

#### LncRNA-MM2P

LncRNA-5730422e09Rik was recognized as the uniquely upregulated LncRNA during M2 macrophage polarization. It is renamed as LncRNA-MM2P (LncRNA-macrophage M2 polarization) because of its effects on M2 macrophages (Refs 85, 111). Accordingly, a study performed in 2019, investigated the effect of LnRNA-MM2P on regulation of TAM/M2 polarization. It started with monitoring the existence of LncRNA-MM2P in HCC samples, which was positively confirmed. Then, three factors, which were known to have effect on M2 polarization, were put into consideration: macrophages, IL-4 and IL-13. Knocking down of LncRNA-MM2P was accomplished by using two siRNA sequences, followed by IL-13 and IL-4 induction. Results showed that LncRNA-MM2P is vital for IL-4 and IL-13 that in turn induced M2 polarization.

Unlike M1 macrophages, LncRNA-MM2P has no effect on their polarization. Then the study focused on the functions of M2 in respect to LncRNA-MM2P including angiogenesis, tumourigenesis and phosphorylation of STAT. Regarding angiogenesis, collective data revealed that knocking down of LncRNA-MM2P abolishes angiogenesis-promoting feature of M2 macrophages and subsequently inhibits tumourigenesis and tumour growth.

Along with the previous results, LncRNA-MM2P was found to regulate de-phosphorylation of STAT-6 contributed to M2 macrophage polarization in response to IL-13 or IL-4 (Ref. 85).

All the above results support the idea that LncRNA MM2P is an important positive regulator for M2/TAM polarization and functions (Table 1).

#### LncRNA MIAT

LncRNA MIAT (myocardial infarction associated transcript) or RNCR2 (retinal non-coding RNA 2) (Ref. 112) has been correlated with various diseases including cancer (Refs 113, 114, 115). Many studies performed on LncRNA MIAT suggested that it indirectly regulates macrophages' polarization and functions. Two complementary studies investigated the role of MIAT in M2 polarization in HCC; one study reported the presence of LncRNA MIAT in HCC and its' effects on some factors that have roles in macrophages' polarization and occurrence. However, the second study correlated one of the reported factors that were found to be regulated by LncRNA MIAT and inspected its' effects on macrophages.

The first study was monitoring the effect of LncRNA MIAT in HCC, as LncRNA MIAT was found to be upregulated in HCC cell lines. The outcome showed that MIAT wasn't only inversely related to the expression of miR-214, but also upregulation of MIAT was directly associated with high level of histone H3 and H4 acetylation in HCC tissues compared with normal tissues. Moreover, when MIAT was upregulated; HCC cell viability, proliferation and invasion were all enhanced. All these effects occurred as a result of MIATs' negative regulation on miR-214. miR-214 is a well-known tumour-suppressor agent that restrains the proliferation and invasion of HCC cells (Ref. 86).

The second one reported that miR-214 is downregulated in TAMs in HCC tissues. In this study, the hypothesis that miR-214 only plays a role in suppression of cell growth and cell invasion of HCC was eliminated. miR-214 presented a role in repolarization of M2 into M1 phenotype. miR-214 performs this function through inhibiting the *β*-catenin signalling pathway. The *β*-catenin signalling pathway inhibits polarization of macrophages into M1 and shifts the polarization towards TAMs/M2 (Ref. 116).

Collectively, all the above results suggest that LncRNA MIAT indirectly affect M2/TAM polarization and functions including cell growth and invasion. It achieves this aim through two main pathways downregulation of miR-214 that sequentially enhance the *β*-catenin signalling pathway (Table 1).

### Transcribed ultra-conserved regions

Transcribed ultra-conserved regions (T-UCRs) are a special class of ncRNAs. T-UCRs are highly suggested to contribute to oncogenic pathways. Recent studies showed that T-UCRs contribute to various cellular pathways, such as DNA damage response, proliferation, chemotherapy response, MYCN (v-myc myelocytomatosis viral related oncogene) amplification, gene copy number and immune response and survival rates in some types of cancers. In addition, it was reported that T-UCRs are involved in various types of cancers such as HCC, neuroblastoma, bladder cancer, pancreatic cancer, lung cancer, prostate cancer, breast cancer, gastric and colon cancers (Ref. 117).

#### T-UCR uc.306

The expression of uc.306 was found to be significantly downregulated in HCC cells relative to normal cells. In addition, the relative expression of T-UCR uc.306 was higher in M1 than in M2 macrophages. It is revealed that uc.306 is involved in different pathways including the Wnt pathway, Hippo signalling pathway and Hedgehog signalling pathway, which are known to affect M2/TAM polarization by different ways. It is concluded that uc.306 plays a role in HCC development by affecting HCC formation, growth, migration, metastasis and EMT. Therefore, it is suggested that uc.306 has a relation with TAMs/M2, since all these pathways affect M2 polarization, filtration and function. It is very clear that further research in the exact targets of uc.306 on macrophages in different cancers including HCC is of great importance, as this collective data suggest that uc.306 can be a novel marker for the treating and diagnosing HCC (Ref. 87) (Table 1).

### MicroRNAs

Lin-4 was the first discovered microRNA (miRNA) in 1993; before that miRNAs were considered as protein coding regions (Ref. 118). miRNAs are small non-coding RNAs, with a length of an average 22 nucleotides. Mostly DNA sequences are transcribed into primary miRNAs and sort out into precursor miRNAs and finally into mature miRNAs. miRNAs can control 30–90% of post-transcriptional regulation of gene expression through different mechanisms; interaction with one of these regions: the 3′ untranslated region (UTR) of target mRNAs, coding sequence or gene promoters (Refs 118, 119). Moreover, miRNAs are important in normal cell development and are involved in various biological processes (such as cell growth, activation, apoptosis and differentiation) (Ref. 120). In addition, miRNAs have a vital role in cell–cell communication as they act as signalling molecules. Since miRNAs are secreted in the extracellular fluids and related to different diseases; miRNAs are potential targets and biomarkers for different diseases including cancers (Refs 120, 121). Various miRNAs were widely studied in HCC; the studies showed that miRNAs have huge roles in HCC progression and survival. Our research group investigated the role of several miRNAs in HCC such as: (1) the common pathway of the opposing miRNAs ‘miR-194-5p and miR-155-5p’ that showed to have the same impact on PD-1/PD-L1 immune checkpoint in HCC (Ref. 122). (2) miR-615-5p that function as a unique tumour suppressor in HCC via two altered mechanisms. miR-615-5p is inversely correlated with insulin-like growth factor-II (IGF-II), that displays significant decrease in cell proliferation and migration upon its forced expression (Ref. 123). In addition, miR-615-5p suppresses IGF type-1 receptor (IGF-1R) in HCC, that in return repress NK cells cytotoxicity (Ref. 124). (3) miRNA-17-5p have the ability to increase IGF-II bioavailability by both targeting and suppressing insulin-like growth factor binding protein-3 (IGFBP-3) expression (Ref. 125). (4) The effects of miRNA-155 were investigated versus three members of the IGF axis (IGF II, IGF-1R and IGFBP-3) were investigated. The results revealed that with miR-155 induction in HCC cell lines, upregulation of IGF-II and IGF-IR and the downregulation of IGFBP-3 can be achieved (Ref. 126). (5) miR-let-7a was studied with respect to different factors ‘DNA methylation, the oncogenic IGF-signalling pathway (specifically IGF2BP-2 and 3)’. The findings revealed that miR-let-7a can be suppressed by DNA hyper-methylation, that in return prompts the oncogenic IGF-signalling pathway (Ref. 127). (6) miRNA-486-5p found to suppress IGF-1R and downstream mammalian target of rapamycin (mTOR), STAT3 and c-Myc enhances in HCC playing a role in tumour suppression (Ref. 128) (Table 2).

**Table 2.** Examples of miRNAs that may affect TAMs epigenetically

miRNA	Model	Main findings	Effect on cancer growth	Ref.
miR-194-5p and miR-155-5p	In silico then in vitro in HCC cells	miR-194-5p and miR-155-5p showed the same impact on PD-1/PD-L1 immune checkpoint in HCC.	Negative	122
miR-615-5p	In silico and in vitro in HCC cells	Acts as a unique tumour suppressor in HCC via two altered mechanisms. Inversely correlated with IGF-II, that displays significant decrease in cell proliferation and migration upon its forced expression.	Negative	123
miR-615-5p	In vitro on HCC cells	Forced the expression of miR-615-5p, repressed IGF-IR, attenuated NK cytotoxicity and decreased CD56^dim^. Increased CD56^bright^ NK subsets and reduced the cytotoxic markers NKG2D, TNF-*α* and perforins. Repressed NKG2D ligand (ULBP2) in Huh-7 cells.	Positive (repress NK cells cytotoxicity)	124
miRNA-17-5p	In silico and in vitro on HCC cells	Increased IGF-II bioavailability by both targeting and suppressing IGFBP-3 expression.	Regulates IGF-II bioavailability and HCC progression	125
miRNA-155	In vitro on HCC cells	oncomiR, which upregulates the oncogenes, IGF-II and IGF-IR, and downregulates the tumour suppressor, IGFBP-3. Increased HCC cell carcinogenicity.	Positive	126
miR-let-7a	In vitro on HCC cells	Mimics diminished IGF-II as well as IGF2BP-2/-3 expression. DNA hyper-methylation leads to epigenetic repression of miR-let-7a in HCC cells, which induces the oncogenic IGF-signalling pathway.	Negative	127
miRNA-486-5p	HCC tissues and Huh-7 cells	Acts as a tumour suppressor in HCC through the repression of essential members of the IGF-axis, including IGF-1R and its downstream mediators mTOR, STAT3 and c-Myc.	Negative	129

Moreover, miRNAs were studied in macrophages specifically in TME of solid tumours such as HCC. The results showed the great effect of miRNAs on macrophage polarization and functions. Some of these examples will be discussed in this section.

#### microRNA 98

MicroRNA 98 (miR-98) is an RNA gene that is associated with various diseases including non-small cell carcinoma lung cancer (Ref. 130) and HCC (Ref. 88). Diverse pathways are related to miR-98, such as Wnt/*β*-catenin signalling pathway (Ref. 131) and TLR signalling pathway (Ref. 132).

miR-98 has been studied in HCC and showed that it has a vital role in tumour suppression in HCC because of its effect on two different pathways. The first pathway is the Wnt/*β*-catenin signalling pathway. miR-98 is a member of let-7 family, that work on suppressing the Wnt/*β*-catenin signalling pathway. Wnt/*β*-catenin signalling pathway is mainly responsible for macrophage polarization. The second pathway is targeting the enhancer of Zeste homologue-2 (EZH2) that can cause downregulation of multiple tumour suppressors, thus promoting liver cancer metastasis. Regarding M2 specifically, miR-98's expression was measured in all macrophages' forms (M1/M2/MO) in HCC cell lines. The findings attested that miR-98 is downregulated in M2/TAMs unlike M1. These results were observed after measuring the secretions of both M1 (TNF-*α* and IL-1*β*) and M2 (TGF-*β* and IL-10). Later, the effect of miR-98 on TAM functions including migration, invasion and EMT was investigated through knockdown of miR-98 in TAMs. The findings revealed that the expression of miR-98 regulates migration, invasion and EMT through modulating these effects (Ref. 88) (Table 1).

#### miRNA 125a/b and miR-99b

To the best of our knowledge several studies were concerned with miR-125, but unfortunately very few were concerned with its role in HCC. Two studies were concerned with the role of both miRNAs in HCC.

The first study reported that both miRNA125a and miRNA125b are downregulated in the exosomes of HCC associated with M2/TAMs, when compared with normal cells. miR-125a/b can target CD90, that in return can suppress HCC cell growth and sphere formation, that was observed after being assayed by CCK8. In addition, it was observed that miRNA125a/b inhibits TAMs mediated in cancer stem cells of HCC by targeting CD90 as well (Ref. 89).

The second study investigated the relation between both miRNAs and regulation of HCC progression and macrophage polarization. In a previous study, it was demonstrated that miR-125 has effects on macrophages in HCC and that miR-99b can regulate myeloid cell differentiation and macrophage activation. Since both miR-99b and miR-125a belong to one miRNA cluster that often manage their role in cell differentiation, it was proposed that miR-99b and miR-125a could regulate macrophage polarization as a cluster. In this study, it was found that overexpression of miR-99b promotes the differentiation of monocytes into macrophages rather than granulocytes (under GM-CSF stimulation). Furthermore, overexpression of miR-99b in macrophages promoted M1 polarization by targeting *κ*B-Ras2 and mTOR, simultaneously inhibiting M2 polarization via mTOR/IRF4. However, it was reported that the capacity of miR-99b-mediated M1 macrophage polarization and function was almost equal to that of the miR-99b–miR-125a cluster, and more than that of miR-125, suggesting that miR-99b may be an important target in regulating M1 macrophage polarization and function. When miR-99b and miR-125a were delivered by nanoparticles to the tumour, tumour growth was restricted through repolarization of M2/TAMs to M1 followed by immunosuppressive microenvironment (Ref. 90) (Table 1).

Summing up, both miR-125 and miR-99 are considered tumour-suppressor reagents. They are downregulated in different cancers including HCC. Targeting these miRNAs can be a successful approach in order to control TAM polarization that in turn affects tumour progression.

### Histone modulations and polarization of TAMs

Histone modification is a post-translational modification that tackles histone proteins. Different modifications can occur including methylation, phosphorylation, acetylation, ubiquitylation and sumoylation. These modifications can influence gene expression by altering chromatin structure or recruiting histone modifiers. Hereby, we list a group of mechanisms of epigenetic histone modulations (Table 1) responsible for polarization of TAMs in different types of cancers.

According to Tikhanovich *et al.* (Ref. 91), the livers of cirrhosis patients with a history of recurrent infections showed abnormalities in protein arginine methyltransferase 1 (PRMT1) activity and arginine methylation. After caecal ligation and puncture, mice lacking PRMT1 produce more proinflammatory cytokines and have worse survival rates. Defective (peroxisome proliferator-activated receptor (PPAR))-dependent M2 macrophage differentiation is the source of this impairment. In addition, PPAR expression was four-fold lower in PRMT1 knockout cells compared with wild-type cells as a result of their inability to upregulate it in response to IL-4 treatment. Through histone H4R3me2a methylation at the PPAR promoter, PRMT1 controls the expression of the PPAR gene. Upon the administration of Rosiglitazone and GW1929 they restored M2 differentiation both in vivo and in vitro, eliminating the difference in survival between PRMT1 knockout and wild-type mice. All of these findings point to a role for PRMT1 in the control of macrophage PPAR expression, which may help explain why infection susceptibility is increased in PRMT1 knockout mice (Ref. 133).

Another study was conducted about the role of PRMT1 in the pathogenesis of atherosclerosis through MHC II transactivation. It indicated the interactions between class II transactivator (CIITA) and PRMT1. PRMT1 expression was downregulated by IFN-*γ* treatment, and PRMT1 binding to the MHC II promoter was diminished. Although PRMT1 depletion increased MHC II transactivation, PRMT1 overexpression suppressed MHC II promoter activity. PRMT1 increased CIITA degradation by methylation. Therefore, these results demonstrate that PRMT1 has a recently unknown function in inhibiting CIITA-mediated MHC II transactivation (Ref. 134).

It worth noting that the role of chromatin modifiers in oncogenesis has been a fascinating area of cancer study. These are the enzymes that post-translationally alter chromatin through processes including methylation, acetylation, sumoylation and phosphorylation, among others. Chromatin modifiers can either promote or inhibit transcription, depending on the change. A chromatin modification known as Su(var)3-9, enhancer-of-zeste and trithorax (SET) and MYN-domain containing 3 (SMYD3) has been linked to the emergence and spread of several cancer forms. Histone 3 lysine 4 (H3K4), a methylation mark known to enhance transcription, was tri-methylated for the first time. Other histone (e.g. H4K5 and H4K20) and non-histone (VEGFR, human epidermal growth factor receptor 2 (HER2), mitogen-activated protein 3 kinase 2 (MAP3K2), estrogen receptor (ER) and others) substrates of SMYD3 have, however, been discovered since this finding, especially in relation to cancer (Ref. 135).

In the context of insulin resistance, the findings by Yoshizaki *et al.* (Ref. 93) establish a novel function for sirtuins (SIRT)1 as a key regulator of macrophage inflammatory responses, and they suggest that targeting SIRT1 may be a beneficial approach for treating the inflammatory component of metabolic disorders. They demonstrated that SIRT1 depletion causes a wide activation of the c-Jun N-terminal kinases (JNK) and IκB kinase complex (IKK) inflammatory pathways in intraperitoneal macrophages and the murine macrophage RAW264.7 cell line, as well as an increase in LPS-stimulated TNF-*α* release. Furthermore, gene expression profiles show that SIRT1 knockdown increases the expression of inflammatory genes. We further show that SIRT1 activators reduce LPS-stimulated inflammatory pathways and TNF-*α* release in RAW264.7 cells and primary intraperitoneal macrophages in a SIRT1-dependent manner. Using a SIRT1 activator to treat Zucker fatty rats results in significant improvements in glucose tolerance, decreased hyperinsulinaemia and increased systemic insulin sensitivity. These in vivo insulin-sensitizing effects were accompanied by a decline in tissue inflammation markers and a reduction in the proinflammatory state of adipose tissue macrophages, which is entirely consistent with SIRT1's actions on macrophages in vitro (Ref. 93).

A study by Li *et al.* showed that downregulation of SIRT4 in TAMs regulates macrophage alternative activation and aids in the development of HCC through the fatty acid oxidation (FAO)–PPAR–STAT3 axis. These findings could offer a fresh therapeutic focus for the treatment of HCC. SIRT4 expression in peritoneal tissues was positively correlated with patient survival and dramatically downregulated in HCC tumour. Using gene interference, they discovered that inhibiting SIRT4 in TAMs drastically modified macrophage alternative activation and enhanced HCC cell proliferation both in vitro and in vivo. Moreover, they identified the mechanism through which HCM limited SIRT4 expression in macrophages and encouraged alternative macrophage activation through the FAO–PPAR–STAT3 axis. Additionally demonstrated that enhanced SIRT4-induced SIRT4 downregulation was the cause of increased TAM infiltration in peritumour tissues (Ref. 94).

A study by Mullican *et al.* (Ref. 95) proved that macrophages missing histone deacetylase 3 (HDAC3) exhibit a polarization phenotype resembling IL-4-induced alternative activation and are also hyperresponsive to IL-4 stimulation. HDAC3 deacetylates histone tails at regulatory sites across the macrophage genome, repressing several IL-4-regulated genes that are indicative of alternative activation. Pulmonary inflammation was reduced in animals missing HDAC3 in macrophages after exposure to *Schistosoma mansoni* eggs, a model of Th2 cytokine-mediated illness that is restricted by alternative activation. HDAC3 thus performs alternate activation as a brake whose release may be advantageous in the therapy of a variety of inflammatory disorders (Ref. 95).

Furthermore, myeloid Hdac3 loss promotes collagen deposition in atherosclerotic lesions and hence creates a persistent plaque phenotype using conditional knockout mice. Additionally, macrophages demonstrated an improvement in lipid processing and a flip to anti-inflammatory wound healing features. After Hdac3 was eliminated, the Tgfb1 locus underwent epigenetic regulation, which caused smooth muscle cells to produce more collagen, resulting in the pro-fibrotic phenotype. Additionally, HDAC3 was the only HDAC in humans that was increased in ruptured atherosclerotic lesions, HDAC3 expression was negatively connected with pro-fibrotic TGFB1 expression, and HDAC3 was associated with inflammatory macrophages. Overall, modifying the macrophage epigenome can enhance the course of atherosclerosis, and identify HDAC3 as a possible new therapeutic target for cardiovascular disease (Ref. 136).

According to reports, specific inhibition of HDAC6 slows tumour development in a variety of cancers. It is still unclear, though, which cellular elements are causing this action. A study assessed the HDAC6i Nexturastat A as a priming agent to assist the change from a ‘cold’ to a ‘hot’ TME and may enhance immunological check-point blocking therapy. In syngeneic melanoma tumour models, this combination of modalities has been shown to dramatically slow tumour development. In addition, we found that anti-PD-1 blocking medication completely neutralized the upregulation of PD-L1 and other immunosuppressive pathways. Additionally, this combination demonstrated substantial modifications in the TME, including improved immune cell infiltration, increased central and effector T cell memory and a notable decline in tumour-promoting M2 macrophages. According to the analysis of the different elements of the TME, HDAC6i in vivo anti-tumour action is mediated through its impact on tumour cells and TAMs rather than directly by T cells. Overall, these findings suggest that selective HDAC6i might be employed as immunological priming agents to arouse immunologically ‘cool’ tumours, which would then enhance ongoing immune checkpoint blocking therapy (Ref. 96).

### Chromatin remodelling

Chromatin represents a dynamic structural entity comprising DNA and histone proteins, notably H2A, H2B, H3, H4 and DNA itself. The conformation of chromatin is intricately regulated by DNA modifications and post-translational modifications of histones, thereby exerting influence over transcriptional activity (Refs 137, 138, 139, 140). The SWI/SNF (switch/sucrose non-fermenting) complex, which operates in an adenosine tri phosphate (ATP)-dependent nucleosome remodelling manner, stands out as the extensively investigated chromatin remodelling complex. Consequently, the genomic region encoding of this complex is frequently mutated under neoplastic conditions (Ref. 141). The SWI/SNF complex is a complex macromolecular assembly comprising 12–15 subunits, prominently featuring a catalytic ATPase subunit known as SWI/SNF-related, matrix-associated, actin-dependent regulator of chromatin, subfamily A, member 4 (SMARCA4). Other subunits include adenosine thymine (AT)-rich interaction domain 1A (ARID1A), ARID1B, polybromo 1 (PBRM1) and ARID2 (Ref. 142). In HCC, mutations that inactivate SWI/SNF subunits, including ARID1A, ARID1B, ARID2, PBRM1 and SMARCA4, are frequently observed (Ref. 141).

ARID1A, a subunit of the SWI/SNF complex, is crucial for DNA accessibility in processes such as transcription, DNA repair and replication through chromatin remodelling. ARID1A mutations are linked to larger and moderately differentiated HCC tumours, metastasis and poor prognosis (Refs 143, 144). These mutations also promote angiogenesis through H3K27ac modification. In mice, ARID1A knockdown induces hepatocarcinogenesis with immune cell infiltration and pathway activation (Ref. 145). Mutations in the SWI/SNF complex, including ARID1A, contribute to resistance to immune checkpoint inhibitors, with ARID1A-deficient tumours showing better responses to anti-PD-L1 treatment. Inhibiting another SWI/SNF subunit, PBRM1, enhances immunotherapy responses by boosting tumour immunogenicity (Refs 146, 147). These findings suggest targeting the SWI/SNF complex may enhance the effects of immune checkpoint inhibitors in HCC, though more research is needed to understand the mechanisms involved (Ref. 148).

### RNA modifications

RNA modification plays a pivotal role as a post-transcriptional gene expression regulator (Ref. 149). Recent advancements in molecular and sequencing technologies have significantly boosted the investigation of RNA modification. Growing evidence highlights the significance of RNA modification dysregulation in the pathogenesis of various human diseases, with a particular focus on HCC (Ref. 150). Eukaryotes exhibit a range of cancer-related RNA modifications, including *N*6-methyladenosine (m6A), *N*1-methyladenosine (m1A), 5-methylcytosine (m5C), 2′-*O*-methylation, *N*7-methylguanosine, pseudouridylation, adenosine-to-inosine and 5-methoxycarbonylmethyl-2-thiouridine (Refs 151, 152). Among these, m6A is the most extensively investigated and comprehensively characterized (Ref. 153).

For instance, KIAA1429 has been shown to enhance HCC invasiveness and migration by modifying m6A methylation in ID2 and GATA3 mRNA (Ref. 154). ALKBH5 has been demonstrated to suppress HCC proliferation and invasion by regulating m6A-mediated epigenetic inhibition of LYPD1 (Ref. 155), whereas METTL3 promotes HCC progression through post-transcriptional silencing of SOCS2 (Ref. 156). Various other RNA methylations have also been associated with HCC. For instance, the aberrant NSUN2-mediated m5C modification of H19 LncRNA has been linked to poor HCC differentiation (Ref. 157). Additionally, TRMT6/TRMT61A-mediated m1A methylation has been shown to be essential for the self-renewal of liver cancer stem cells and tumourigenesis (Ref. 158). Recent investigations have started examining interactions within specific RNA modification types. For example, Fang *et al.* (Ref. 147) developed a two-m6A gene-based signature (HNRNPA2B1 and RBM15) predicting HBV-related HCC prognosis (Ref. 159). Similarly, a risk signature involving four m1A regulators (TRMT6, TRMT61A, TRMT10C and YTHDF1) strongly correlated with HCC patient prognosis (Ref. 160). In summary, although RNA modifications and the altered expression of related regulatory genes have been investigated in various aspects of HCC and its development, their exact functions and impacts on tumourigenesis, proliferation, metastasis and resistance demand further in-depth exploration and analysis.

### DNA methylation

To date, DNA methylation effects on macrophage polarization in HCC specifically are not fully understood. Therefore, further research is needed to figure out the specific mechanisms that DNA methylation follows in order to modulate macrophages' polarization and functions. In this section, some DNA methylation modifications that modulate the polarization of macrophages are discussed.

DNMT3B is one of the well-known DNA methyltransferase (DNMT) that has a role in M2 differentiation and phenotypic control. It encodes DNA methyl transferase 3 beta. Generally, any alteration in this gene is associated with immunodeficiency. When DNMT3B is methylated, this negatively affects M2/TAM polarization. By modifying DNMT3B, M2 polarization was promoted unlike M1 polarization that was inhibited. Moreover, overexpression of DNMT3B downregulates IL-4-induced expression of Arg-1. Downregulation of IL-4 causes methylation in the promoter of PPAR*γ*, contributing to downregulation of macrophage polarization (Ref. 97).

Another example for DNA methylation that can affect M2/TAM polarization is methylation of oxidored nitro domain containing protein 1 (NOR1). NOR1 is over expressed in HCC tissues. It is suggested that NOR1 promotes M2 polarization. NOR1 was found to be silenced by hyper-methylation that can affect M2 polarization and associated with poor prognosis of the disease because of increased expression of iNOS. Nonetheless, it decreased expression of Arg-1, Ym1 and IL-10 mRNA in Kupffer cells (KCs) from NOR1-KO mice (Ref. 98).

### Proteomics

Another turning point in TAM studies is protein regulation in TAMs. Some proteins when overexpressed they enhance the density of TAMs in the tumour and TME; for instance two are discussed below.

#### Yes-associated protein (YAP)

YAP is a transcriptional regulator that acts on the genes concerned with cell proliferation and apoptosis. Studies showed that activation of YAP is an important step in TAM recruitment towards TME in HCC via modulating the levels of IL-6, CSF-1 and CCL-2 secreted by the tumour cells, thereby inducing the formation of tumour initiation cells and remodelling the composition of TME. In addition, it was found that YAP also affects the polarization of macrophages, as silencing YAP caused a decrease in M2 levels and other polarization-related factors (such as *β*-catenin, Akt and NF-*κ*B), but no change was monitored in M1 (Refs 99, 161) (Table 1).

#### MYC

It has been recognized as a key factor in M2 macrophage activation. Silencing of MYC gene in macrophages showed a decrease in tumour angiogenesis and reduction in tumour growth. MYC is known as a transcription factor, which promotes cell growth and differentiation. MYC likewise can control the expression of the tumour-promoting factors: matrix metallopeptidase 9 (MMP9), VEGF, TGF-*β* and HIF-1 (Ref. 100). Moreover, MYC in macrophages increases the expression of M2-specific genes such as ALOX15, MRC1 and SCARB1. Therefore, MYC can control M2 polarization (Ref. 162). Recent studies have investigated effect of MYC along with TWIST protein. The studies showed that they both work together in order to promote metastasis, recruitment and polarization of macrophages through evoking cytokines release, including CCL2 and IL-13, to facilitate crosstalk between cancer cells and host macrophages that promotes tumour progression (Ref. 101).

### Signalling pathways involved in TAM polarization

Different pathways are associated with macrophage polarization, recruitment and functions. Mostly the above epigenetic factors affect one or more of these pathway(s) that in return affect M2/TAM functions and polarization. In this section, some of these pathways are discussed.

#### Wnt/*β*-catenin pathway

Wnt ligands have significant roles in cellular migration, proliferation and tissue patterning during embryological development. Recently, studies have found that Wnt ligands are also involved in various diseases specially cancers including HCC. When Wnt ligands are secreted into the extracellular area, they bind to Frizzled receptors on the signal-competent cells to induce the canonical Wnt/*β*-catenin signalling or non-canonical Wnt/Ca2+ signalling pathways. In liver, Wnt ligands and their receptors are expressed on different hepatic cell types (such as hepatocytes and KCs). A study which investigated the effect of the Wnt/*β*-catenin pathway on macrophages revealed that Wnt/*β*-catenin signalling (1) was activated during macrophage differentiation (2) greatly expressed in M2-polarized macrophages and (3) enhanced M2 macrophage polarization through c-Myc (Ref. 101) (Table 1).

#### STAT-6

TAMs are regulated indirectly by the STAT-6 pathway as this pathway is a signalling pathway for both cytokines IL-4 and IL-13, which are related to TAMs' polarization. When these cytokines are released in TME, they directly bind to their receptors IL-4R*α* and IL-13R*α*1. This binding results in the activation of the Jak/Stat pathway (phosphorylation of STAT-6), which in turn leads to translocation of pSTAT-6 to the nuclei. This translocation causes activation of the transcription of target genes that are specific for M2 macrophages, including mannose receptor 1 (*Mrc-1*), resistin-like *α* (*Retnla*, *Fizz1*), chitinase 3-like 3 (*Chi3l3*) and chitinase 3-like protein 3 (*Ym-1*) and inhibition of M1-associated signalling pathways (Ref. 102) (Table 1).

#### PI3K/Akt signalling

The PI3K/Akt pathway is one of the pathways that can regulate macrophage migration, polarization and survival. Moreover, this pathway controls the responses to various metabolic and inflammatory signals in macrophages. It has been reported that not only PI3K is a crucial step in M2 polarization in response to surfactant protein A or IL-4 but also activation of AKT is of great importance. Some signals such as TGF-*β*, IL-10 and bone morphogenetic protein 7 (BMP-7) are regulated through PI3K/Akt. These signals are mainly responsible for enhancing M2 polarization. Moreover, Akt activation is crucial in inducing IL-10 in macrophages (that play a role in polarization of M2) through pro-inflammatory signals. Nonetheless, PI3K and Akt isoforms were found to contribute also to M1 polarization (Ref. 101) (Table 1).

### Immunotherapeutic applications of TAM-associated epigenetic modifications and perspective

TAMs represent a force to be taken into consideration for effective cancer therapy as intratumourally infiltrating immune cells with the highest number (Ref. 51). Nowadays, multiple treatment strategies have been reported for targeting epigenetic factors affecting TAMs in TME. The main targets of the epigenetic modifiers are the three groups of enzymes known as readers, writers and erasers. Tyrosine kinases, serine-threonine kinases, DNMT and enzymes such as histone acetyltransferases and histone lysine methyltransferases are examples of the group of enzymes known as epigenetic writers that add methyl or acetyl groups to histone proteins. Readers are proteins that detect functional modifications of epigenetic marks placed on DNA or histones with binding domains for covalent modifications, such as bromodomains involved in histone acetylation, chromodomains involved in histone methylation and methyl CpG-binding proteins, which allow conformational modulation of the chromodomain via dynamic integrated signals (Ref. 163). HDACs and histone demethylases are examples of erasers, which are enzymes that remove epigenetic changes from histone proteins (Ref. 164). DNMTs and HDACs are reported to influence TAMs in TME as previously mentioned. Regarding HCC, guadecitabine, a small-molecule inhibitor, is undergoing clinical trial stage II (NCT01752933) as a DNMT inhibitor for sorafenib-resistant patients in advanced stage (https://clinicaltrials.gov/study/NCT01752933).

A study demonstrated that systemic injection of DNMTi deacetylase activity (DAC)-induced TAM activation towards an M1-like phenotype in a colorectal cancer model. In a DNMTi-independent manner, DAC binds ATP-binding cassette transporter A9 and promoted cholesterol build-up, which boosted p65 phosphorylation and IL-6 expression (Ref. 165). More evidence is needed to validate the results reported in this study on other types of cancers. However, it is widely agreed that DNMTi therapies would improve the immune microenvironment from the point of tumour infiltrating T cells by reactivating the expression of immunosurveillance-related genes in tumour cells. One study reported a combination treatment using DNMTi along with Immune checkpoint inhibitors (ICIs) has a beneficial therapeutic effect on pancreatic cancer (Ref. 166). More studies investigating the combination of epigenetic modulators along with other immune therapies need to be conducted.

Infusing M1 macrophages has been investigated in orthotopic pancreatic cancer models. The study reported that infusion of M1 macrophages alone increased distal metastasis and depleted endogenous macrophages, because M1 macrophages would be transformed to TAMs once they infiltrated TME. DNMTi therapy of infused macrophages, on the contrary, suppressed TAM metabolic processes and dramatically reduced metastasis (Ref. 167). Despite the fact that the long-term impacts of DNMTi could not be examined because of the short trial length, this work provides significant evidence for the possible use of epigenetically fortified macrophages in treatment of other cancer types.

Phenotypic instability of the macrophages in the TME could be used to lock them in M1 state where it has been suggested that suppressing some factors, such as TET2 and PRMT2, could delay M2 polarization, whereas exogenous production of a number of epigenetic regulators, such as DNMT1 and DNMT3B, may accelerate the M1 polarization of macrophages. Today, powerful tools such as CRISPR-Cas9 and PROTAC technologies are available for targeted gene editing and managed protein degradation (Refs 168, 169).

Multiple studies highlighted a connection between immune checkpoint proteins and TAMs in TME. It is well established that the activation of programmed cell death-1 and its ligand (PD-1/PD-L1) immune checkpoint leads to T cell anergy and facilitates immune evasion (Ref. 170). Two studies have reported that TAMs express PD-1 and that was associated with poor prognosis of cancer along with increase in metastasis as a result of a decrease in macrophage phagocytosis. M2 macrophages were the predominant reported phenotype (Refs 170, 171). These results suggest that PD-1/PD-L1 treatments may depend on macrophages mechanistically, which has considerable consequences for HCC treatment and explains the current combinational approaches in clinical trials. Belinostat, a HDAC inhibitor, has been shown to enhance the anticancer effects of anti-CTLA-4 therapy in a HCC mouse model with increased IFN generated by anti-tumour T-cells and decreased regulatory T cells (Ref. 172). Another combinational therapy between guadecitabine and durvalumab (anti-PD-L1 monoclonal antibody) is under investigation in phase I (NCT03257761) for recurrent HCC patients (https://classic.clinicaltrials.gov/ct2/show/NCT03257761). Additionally, one study reported that TAM infiltration into the HCC TME was enhanced by miR-148b deficiency, leading to disease progression and metastasis (Ref. 172). Furthermore, increased TAM infiltration led to PD-L1 overexpression in HCC cells via the NF-*κ*B/STAT3 pathway (Ref. 172). Accordingly, inhibiting the osteopontin/CSF1/CSF1R signalling pathway could convert TAMs from M2 to M1 and reduce PD-L1 expression, improving response to anti-PD-1/PD-L1 therapy in HCC mouse models (Ref. 173).

TAM infiltration and polarization key regulators could be targeted as an immunotherapeutic approach for HCC. For example, *Listeria monocytogenes*-based tumour vaccine (Lmdd-MPFG) was thought to promote TAM reversal from M2 to M1 phenotypes via TLR2/MyD88-dependent NF-*κ*B activation and p62-mediated autophagy pathway promotion, thus complementing the effects of anti-PD-1 mono-clonal antibodies in HCC immunotherapy (Ref. 172).

Another combinational approach between a natural compound and PD-L1 immunotherapy has been identified.

When combined with anti-PD-L1, cryptotanshinone (CT), a new natural product compound, demonstrated potent anticancer efficacy in Hepa1-6-bearing mice. CT had the ability to promote anticancer M1 polarization via the TLR7/MyD88/NF-*κ*B axis as well as induce an anti-tumour CD8+ response (Ref. 172). However, it is important to consider that MyD88's involvement in TAMs may differ, resulting in M1 or M2 polarization in various situations. MyD88 may have a role in the development of pro-tumour immunity, since therapy with its inhibitor, TJ-M2010-5, resulted in an increase in anti-tumour M1 macrophages (F4/80 CD11c) in the TME and lowered HCC growth (Ref. 172).

Some studies highlighted the inter-connection between TAMs and other immune checkpoints than PD-1/PD-L1. CTLA-4 immune checkpoint is thought to be involved in TAM-mediated HCC immunotherapies. The transcription factor Sal-like protein-4 (SALL4) and miR-146a-5p axis is involved in M2 polarization and increase of expression CTLA-4 and PD-1 on exhausted T cells in HCC (Ref. 174). Targeting this transcriptional axis could have a potential effect on TAM polarization and inhibition of CTLA-4 simultaneously. C-C motif chemokine ligand 14 (CCL14) expression was negatively associated with PD-1, TIM-3 and CTLA-4 expression in HCC where it promoted the infiltration of numerous tumour immune cells, including macrophages leading to poor prognosis (Ref. 174). Surprisingly, injecting nanoliposome-loaded C6-ceramide into HCC mice reduced the number of TAMs, promoting the anti-tumour immunological response of CD8+ T cells (Ref. 175).

TME–TAM interaction therapies would also be effective for epigenetically controlling macrophage activities. Humanized neutralizing antibodies targeting cytokines or receptors contributing to M2 polarization-associated epigenetic changes, such as IL-4 and IL-1, are now available (Ref. 176). Because macromolecular medications tend to concentrate in tumours with aberrant tumour vasculature’, the concentration of these drugs in TME could be raised further with nanotechnology (Ref. 177). Given that cytokines influence more than one type of immune cell, these neutralizing antibodies would significantly reshape the tumour immunological milieu (Ref. 178). Other therapies aimed at suppressing the activity of other cell types in the TME may also have an effect on TAMs. Gefitinib, an epidermal growth factor receptor (EGFR) inhibitor that inhibits tumour growth and angiogenesis, for example, has recently been demonstrated to reduce crosstalk between macrophages and cancer cells by inhibiting receptor-interacting protein kinase 2 (Ref. 179). As the importance of TAMs comes to limelight, more focus will be on investigating macrophage phenotypes through the assessment of cancer therapy.

## Conclusion

HCC incidence has been increasing over the past few years and this rate is expected to increase further in the future. Despite the implementation of advanced treatments strategies, nevertheless, the survival rates of HCC are still low, unlike metastasis and recurrence rates that are still significantly high. Accordingly, accumulating evidence reported some potential factors that may affect HCC progression and survival, including immunity role in tumour progression. Macrophages are type of immune cells that were found to have a direct relation with HCC and cancer progression and survival in general. Macrophages are further polarized whether into M1 or alternatively M2. M1 is a tumour suppression agent unlike M2 that is regarded as an anti-tumour suppressor. M2 is widely considered in most types of cancers as TAMs. TAMs are studied in the area surrounding the tumour called TME versus various epigenetic alterations. LncRNAs, T-UCRs, miRNAs, histone modulations, DNA methylation and some proteins are the epigenetic factors that can contribute to TAM polarization, recruitment and functions. Each of these factors can play a positive or a negative role in TAM occurrence and functions that in return affects the tumour progression and survival. Starting with the LncRNA positive regulators: (1) LncRNA LINC00662 through activation of Wnt/*β*-catenin, (2) LncRNA-TUC339 (3) LncRNA MM2P through regulation of dephosphorylation of STAT-6, (4) LncRNA MIAT through downregulation of miR-214 that in return activate the Wnt/*β*-catenin pathway. Other LncRNAs can play a negative role in TAM existence including: (1) LncRNA cox-2 and (2) LncRNA CASC2c through inhibiting coagulation factor X. T-UCR uc.306 is another positive regulator that acts through more than one pathway including Wnt/*β*-catenin pathway, Hippo signalling pathway and Hedgehog pathway. miR-98 and miR-125a/b and mi-99b are considered negative regulators for TAM polarization and tumour suppressors. miR-125a/b affects the Wnt/*β*-catenin pathway and the EZH2, whereas miR-125a/b and mi-99b have effects on both mTOR/IRF4 and *κ*B-Ras2. Diverse histone modulations can module TAM polarization. DNMT3B and NOR1 are two examples for DNA methylation that affect TAMs. DNMT3B decreases IL-4-induced methylation in the promoter of PPAR*γ* that negatively affect TAM polarization. Although NOR1 works on promoting M2/TAM polarization, some proteins have a crucial role in TAM regulation and recruitment. YAP activation is a crucial step in recruiting TAMs towards TME. In addition, MYC and TWIST are two proteins that work complementarily in the goal of promoting TAM polarization.

This review recommends additional research to fully understand the genetic and epigenetic mechanisms underlying the regulation of TAMs. This in turn could be further related to the clinical stage and phenotype of HCC in the future.
